# Self-Monitoring of Blood Pressure in Hypertension: A UK Primary Care Survey

**DOI:** 10.1155/2012/582068

**Published:** 2011-10-16

**Authors:** S. Baral-Grant, M. S. Haque, A. Nouwen, S. M. Greenfield, R. J. McManus

**Affiliations:** ^1^Primary Care Clinical Sciences, University of Birmingham, Edgbaston, Birmingham B15 2TT, UK; ^2^School of Psychology, University of Birmingham, Edgbaston, Birmingham B15 2TT, UK

## Abstract

This study aimed to determine the prevalence of Self-Monitoring Blood Pressure amongst people with hypertension using a cross-sectional survey. Of the 955 who replied (53%), 293 (31%) reported that they self-monitored blood pressure. Nearly 60% (198/331) self-monitored at least monthly. Diabetic patients monitoring their blood glucose were five times more likely than those not monitoring to monitor their blood pressure. Self-monitoring is less common in the UK than internationally, but is practiced by enough people to warrant greater integration into clinical practice.

## 1. Introduction

Monitoring of blood pressure (BP) is a key aspect of the diagnosis and management of hypertension [1]. Self-monitoring of BP by patients at home is one strategy by which hypertensive patients can participate in their own health care and leads to small but significant reductions in blood pressure [2]. National surveys of adults in the UK show that blood pressure control has gradually improved since the 1990s; however, many patients remain uncontrolled and amongst those at the highest risk, such as those with other comorbid conditions, the situation is worse [3]. Novel interventions are therefore needed if optimum blood pressure control is to be achieved, and self-monitoring appears to be a useful option.

International surveys have found that over 70% of people with hypertension self-monitor blood pressure [4–7]. Available data from the UK suggest much lower uptake in both specialist clinics [8, 9] and the general population [10]. Limited data are available regarding self-monitoring in primary care hypertensive patients.

This study aimed to determine the prevalence of self-monitoring of BP in primary care hypertensive patients and to highlight the characteristics of those that self-monitor blood pressure.

## 2. Methods

A questionnaire was sent to 1815 patients with hypertension registered with four general practices in the West Midlands, UK between November 2008 and April 2009, to determine the prevalence and patterns of use of self-monitoring of blood pressure. Self-monitoring was defined in the questionnaire and information sheet as “taking your own measurements of blood pressure outside your usual visit to your GP practice, usually within the home.” Participating practices were chosen to represent a range of ethnic diversity and affluence of the patient population using the Index of Multiple Deprivation, an estimate of the socioeconomic deprivation of the practice population [11] linked to the practice postcode. Participants were adult patients (18+) identified by Read (morbidity) code with or without a Read code of Diabetes (Type 1 and 2). Patients were requested to return the blank questionnaire if they did not want to participate. A second questionnaire was mailed to nonrespondents approximately two weeks later.

Analyses were undertaken using SPSS (version 15, http://www.spss.com). The results presented are descriptive, reported as percentages and odds ratios with 95% confidence intervals. Demographic characteristics including age, gender, ethnicity, and current status of employment were collected. Some descriptive categories were collapsed for the analysis.

It was assumed that approximately 20% of hypertensive individuals would be self-monitoring BP (twice that seen in a recent UK population survey [10]). To estimate the true prevalence of home self-monitoring with 95% confidence and 5% precision, returned surveys from at least 246 patients were needed. A larger sample drawn from four practices was chosen to increase generalisability and account for nonresponders.

## 3. Results

Of the 1815 questionnaires mailed, 1062 were returned giving a return rate of 59%. Of these, 107 (10%) were returned blank and excluded from analysis, 955 were returned and analysable, giving an overall response rate of 53%. Of these 421/874 (48%) were male, and the age range was 21 to 81+. Of the 931 respondents reporting ethnicity, 81% were white, 6% Asian or Asian British, 7% Black British, 3% were Chinese and 3% were Mixed or other not stated. In view of the small numbers of nonwhite ethnicities, these have been collapsed into one group for the rest of the analysis.

293 reported currently self-monitoring blood pressure (crude prevalence 30.7%, 95% CI 27.8–33.7%). A quarter of respondents (230, 24.1%) had concurrent diabetes, of whom 155 (67.4%) monitored blood glucose and 75 (32.6%) monitored blood pressure. There was no difference in the prevalence of self-monitoring of blood pressure in people with or without diabetes (odds ratio = 1.13, 95% CI 0.82 to 1.55). Amongst the 230 people with diabetes, 156 (68%) monitored blood glucose. This group was 5 times more likely to monitor their blood pressure compared to those that do not monitor their blood glucose (odds ratio = 5.30, 95% CI 2.46 to 11.39). 

Characteristics of those measuring their own BP are shown in Table 1. Younger people (aged between 18 to 60) were 1.5 times more likely to measure their own blood pressure than older people (over 60) (odds ratio 1.48, 95% CI 1.11 to 1.97). The odds of ratio for self-monitoring blood pressure was 1.81 (95% CI 1.27 to 2.59) for nonwhite ethnic group compared to the white ethnic group. Those in employment were also twice as likely to monitor their BP than those not employed (OR = 1.95, 95% CI 1.45–2.63). 

Most people who self-monitored used an automated electronic BP device (247/293, 84.3%; CI 95% 73.5–94.3) with a small percentage (29/293, 9.9%) indicating they monitored using a manual machine. At least 65% reported monitoring at least once per month, most commonly once or twice a week (85/198 43%). Self-reported frequencies are shown in Table 2. Of those respondents currently not self-monitoring, nearly 60% (384/662 58%) reported they would consider self-monitoring in the future.

## 4. Discussion

This survey has found that approximately 30% of primary care patients with hypertension self-monitored blood pressure whether or not they had diabetes. People who self-monitored were more likely to be younger (18–60), in employment (full time or part time), and from minority ethnic backgrounds (Asian, Black, or other ethnic groups) than those who did not self-monitor. People with diabetes who self-monitored blood glucose were more likely to also self-monitor blood pressure.

These findings, in common with those of a local community study [10] support findings from international studies that those with hypertension self-monitor blood pressure more commonly than normotensive populations [4, 6]. The current study suggests that people in primary care self-monitor less frequently than those attending specialist clinics [9], and that despite recent increased marketing of self-monitoring equipment, the UK has some way to go before such monitoring achieves the prominence currently seen internationally [5–7]. One small Scottish study reports that 31% of people with hypertension own a monitor which is similar to our results [12]. Assuming our figures are representative, then over 2 million people with hypertension may be currently self-monitoring in the UK.

In our study the frequency of monitoring for many respondents was low (42% monitoring more than monthly). This may reflect uncertainty of the appropriate frequency of monitoring; in the UK, National guidelines do not specify regimes for self-monitoring of blood pressure other than for diagnosis [13]. Patients and practitioners need better information on which to base self-monitoring regimes. 

The high uptake of self-monitoring in ethnic minority groups could perhaps reflect an increased awareness of the risks of cardiovascular disease amongst this group or by their GPs who may have recommended self-monitoring [14]. An alternative explanation is confounding by age. Our results show that those respondents from minority ethnic groups were younger compared to the white population (as is the case in the population in general) [15] and as self-monitoring was more common in younger people then this may be the explanation. 

The response rate for this study was not as high as hoped and responders may have differed from the rest of the population. However, the proportion of males in the sample (48%, 95% CI 44.9% to 51.5%) was similar to the 2001 census of West Midlands (49%) although the proportion of the people from a White ethnic background (84%, 95% CI 80.9% to 85.7%) was lower than the corresponding 2001 census figure (89%).

The results of this short survey identify a group of individuals with hypertension who currently self-monitor blood pressure with or without GP recommendation. Whilst this could reflect a healthy self-empowered population where hypertensive patients are taking more responsibility for their own health, previous research also suggests that patients may not be reporting this data to their GP or health professional and also monitoring under minimal or no supervision [7, 16]. This could therefore represent a lost opportunity which could be exploited by GPs being aware of the fact that a significant proportion of their hypertensive patients are self-monitoring. 

Self-monitoring is practiced by an appreciable minority in UK primary care. In accordance with the findings from our study, people diagnosed as hypertensive could be potentially three times more likely to self-monitor than the general population. General Practitioners should be aware that around a third of their patients with hypertension could be monitoring their own blood pressure and of the opportunities that this could bring to daily management.

## Figures and Tables

**Table 1:** Characteristics of people self-monitoring and not self-monitoring blood pressure.

Demographics	Self-monitor	Do not self-monitor	Chi-square (*P*)
	*n* ^ a^ (% of total number)	*n* (% of total number)	
Total number	293 (31)	662 (69)	—
Male	137 (50)	284 (47)	0.76 (0.382); NS
Female	135 (49)	318 (53)	
Age range (years)			
18–60	116 (40)	201 (31)	7.13 (0.008)
61 and over	177 (60)	453 (70)	
Ethnic origin			
White	223 (77)	554 (86)	10.98 (0.001)
Other	65 (23)	89 (14)	
Employed*	109 (38)	154 (24)	19.45 (0.001)
Retired/ unemployed	179 (62)	493 (76)	
Antihypertensive medication^a^	261 (90)	579 (88)	0.50 (0.479); NS
Diabetes^+^	75 (25)	155 (23)	0.53 (0.467); NS

^a^Numbers may not add up to total because of missing values.

*Part time or full time employment.

^+^Coded as having diabetes by GP clinical system.

**Table 2:** Self-blood-pressure monitoring frequency reported by self-monitoring respondents (*n* = 305).

	Overall *N* (% of total number)
More than once per day	9 (3)
Once per day	33 (11)
Twice a week	31 (10)
Once per week	54 (18)
Once per month	71 (23)
Not on a regular basis	107 (35)
